# Genetic Variants and Clinical Characteristics of Young‐Onset Parkinson's Disease in the Hakka Population of Western Fujian

**DOI:** 10.1002/brb3.71504

**Published:** 2026-05-27

**Authors:** Li‐Ying Pan, Fang Guo, Chong Zheng, Xiao‐Hong Hu, Yan‐Gui Chen, Rong‐Rong Lin

**Affiliations:** ^1^ Department of Neurology Longyan First Affiliated Hospital of Fujian Medical University Longyan Fujian China; ^2^ Department of Neurology, The Second Affiliated Hospital Zhejiang University School of Medicine Hangzhou Zhejiang China

**Keywords:** clinical features, genetic variants, Hakka population, young‐onset Parkinson's disease (YOPD)

## Abstract

**Research Objective:**

Young‐onset Parkinson's disease (YOPD), defined by symptom onset at or before 50 years of age, has a strong genetic component. The mutation spectra vary markedly across ethnic groups. However, YOPD among the Hakka, a subgroup of the Han Chinese ethnicity, remains uncharacterized. We investigated the genetic and clinical profiles of YOPD in the Hakka population of western Fujian.

**Materials and Methods:**

A total of 33 unrelated patients with YOPD were included in the study. All patients underwent whole exome sequencing (WES) to screen all known Parkinson's disease (PD)‐related genes. Patients with a family history also received a spinocerebellar ataxia (SCA) gene panel test. If the SCA gene panel test result was negative, multiplex ligation‐dependent probe amplification (MLPA) for eight genes including *DJ‐1, ATP13A2, PINK1, UCHL1, SNCA, LRRK2, PRKN*, and *GCH1* was performed. Potential pathogenic variants were confirmed by Sanger sequencing, and both the genetic spectrum and clinical characteristics of patients with YOPD were analyzed.

**Results:**

After variant filtering, six variants in four YOPD‐related genes were identified in four unrelated patients. Of these patients, two harbored pathogenic *ATXN2* repeat expansions. Four variants in *VPS13C* and *PRKN*, along with an *SNCA* exon 1–6 duplication, were classified as variants of uncertain significance (VUS) according to the American College of Medical Genetics and Genomics (ACMG) criteria. A considerable proportion of patients harbored risk variants in *LRRK2*. Furthermore, nine unrelated patients harbored nine variants within six susceptibility genes associated with YOPD, including *EIF4G1*, *COQ2*, *TENM4*, *NR4A2*, *UQCRC1*, and *GBA1*.

**Conclusion:**

This study is the first to analyze the genetic spectrum and clinical characteristics of patients with YOPD in the Hakka population of western Fujian Province. Genetic testing of known pathogenic genes in patients with YOPD can facilitate more accurate diagnosis.

## Graphical abstract

In 33 Hakka young‐onset Parkinson's disease (YOPD) patients from western Fujian, whole exome sequencing (WES) plus spinocerebellar ataxia (SCA) panel testing and multiplex ligation‐dependent probe amplification (MLPA) defined the genetic spectrum. Pathological *ATXN2* repeat expansion was detected in two cases. Four variants in *VPS13C/PRKN* and a *SNCA* exons 16 duplication were variants of uncertain significance (VUS) per American College of Medical Genetics and Genomics (ACMG). Many patients carried *LRRK2* risk variants, and nine unrelated individuals had heterozygous risk variants in *EIF4G1, COQ2, TENM4, NR4A2, UQCRC1*, and *GBA1*.

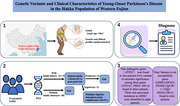

## 1 Introduction

Parkinson's disease (PD) is a common neurodegenerative disorder and is the second most prevalent neurodegenerative disease after Alzheimer's disease (AD) (Alzheimer's Association 2014). The etiology of PD remains unclear, although most studies suggest that it results from a combination of genetic and environmental factors. The pathological features of PD include neuronal inclusions with Lewy bodies and cell loss in the substantia nigra and other brain regions.

The symptoms of PD include classic motor symptoms and nonmotor symptoms. The motor symptoms manifest primarily as bradykinesia, resting tremor, and rigidity, as well as abnormal posture and gait. Nonmotor symptoms include anxiety, depression, constipation, olfactory dysfunction, sensory abnormalities, sleep disturbances, and autonomic dysfunction. As the disease progresses, patients may become disabled, greatly affecting their quality of life. In this study, we chose patients with an onset age of 50 years or younger as young‐onset PD (YOPD) patients, which accounts for approximately 5%–10% of PD cases. Research indicates that patients with YOPD often demonstrate unique clinical manifestations and genetic characteristics and that the disease mechanism is closely related to genetics (Mehanna et al. 2014).

More than 20 genes have been demonstrated to cause or significantly increase the risk of PD (Blauwendraat et al. 2020). In recent years, with advances in gene sequencing technologies, multiple pathogenic genes related to YOPD have been discovered, such as *SNCA, LRRK2*, and *PINK1*, providing new insights into the disease mechanism and the development of targeted therapies.

Notably, studies have shown that the mutation spectrum of YOPD differs among various ethnic groups. For example, the LRRK2 G2019S variant is significantly more frequent in North African Arabs (30%–40%) and Ashkenazi Jews (10%–30%) than in other ethnicities, such as Asian populations (Orr‐Urtreger et al. 2007). In comparison, East Asian patients carry different LRRK2 variants, such as p.I2020T. Additionally, *GBA1* variants are significant risk factors for PD, as observed in East Asian, Southeast Asian, and Indian populations (Koros et al. 2023).

Through whole exome sequencing (WES) and multiplex ligation‐dependent probe amplification (MLPA), a few studies have focused on screening PD‐associated genes primarily in Caucasian populations (Gustavsson et al. 2017; Schormair et al. 2018; Tan et al. 2019). However, large‐scale genetic studies of YOPD cohorts in other populations remain limited (Lin et al. 2019; Zhao et al. 2020). In China, a country with a large population base and a significant aging trend, studies on gene variants related to YOPD are relatively limited. Research has shown that variants in *PRKN* are the most common among Chinese patients with YOPD, accounting for 4.3%–5.7% of all patients, whereas findings regarding the mutation spectrum of other pathogenic genes remain inconsistent (Zhao et al. 2020; Chen et al. 2022; Hua et al. 2022; Sun et al. 2023). This may be related to China's vast territory, differences in the populations studied, and the unique gene mutation profiles specific to different groups.

The Hakka people, a branch of the Han Chinese ethnic group (Parish and Whyte 1980), migrated from the ancient Central Plains (now Henan Province) to southeastern China in approximately AD 300 to escape wars and natural disasters (Luo et al. 2019). The Hakka speak their own distinctive dialect and possess a rich heritage of customs, beliefs, and other cultural elements (Au et al. 2008). In China, the Hakka are distributed primarily in southwestern Fujian, southern Jiangxi, western Guangdong, southeastern Guangxi, Hainan, and Taiwan (Constable 1994). The western Fujian region, especially many counties in Longyan City, is an important settlement area for the Hakka people. There are no systematic reports on the clinical and genetic profiles of YOPD patients in this region.

The main purpose of this study is to analyze the clinical features and genetic variants of patients with YOPD in the Hakka population of western Fujian Province, deepening the understanding of the disease in this specific group.

## 2 Materials and Methods

### 2.1 Participants

From April 2017 to April 2024, patients with PD with an age of onset younger than 50 years were recruited from the Longyan First Affiliated Hospital of Fujian Medical University. All study subjects underwent clinical examinations conducted by two neurologists. All patients met the following inclusion criteria: (1) fulfillment of the revised PD diagnostic criteria established in 2015 by the International Parkinson and Movement Disorder Society (MDS) (Postuma et al. 2015), with classification as either clinically established or clinically probable PD; (2) age of onset ≤50 years; and (3) complete and confirmed familial relationship data via clinical inquiry for cases with positive family history, where a positive family history is defined as the diagnosis of PD in other affected family members being confirmed by two professional neurologists through medical history collection and clinical physical examination. This study was approved by the Ethics Committee of the Longyan First Affiliated Hospital of Fujian Medical University (Approval No. [2021]014). The study was registered in the National Medical Research Registration and Filing Information System of China (https://www.medicalresearch.org.cn) on November 23, 2021, with the registration number MR‐35‐22‐016617. Written informed consent was obtained from all participants or their legally authorized representatives for participation in the study and for the publication of their clinical details.

### 2.2 Research Methods

**FIGURE 1:**
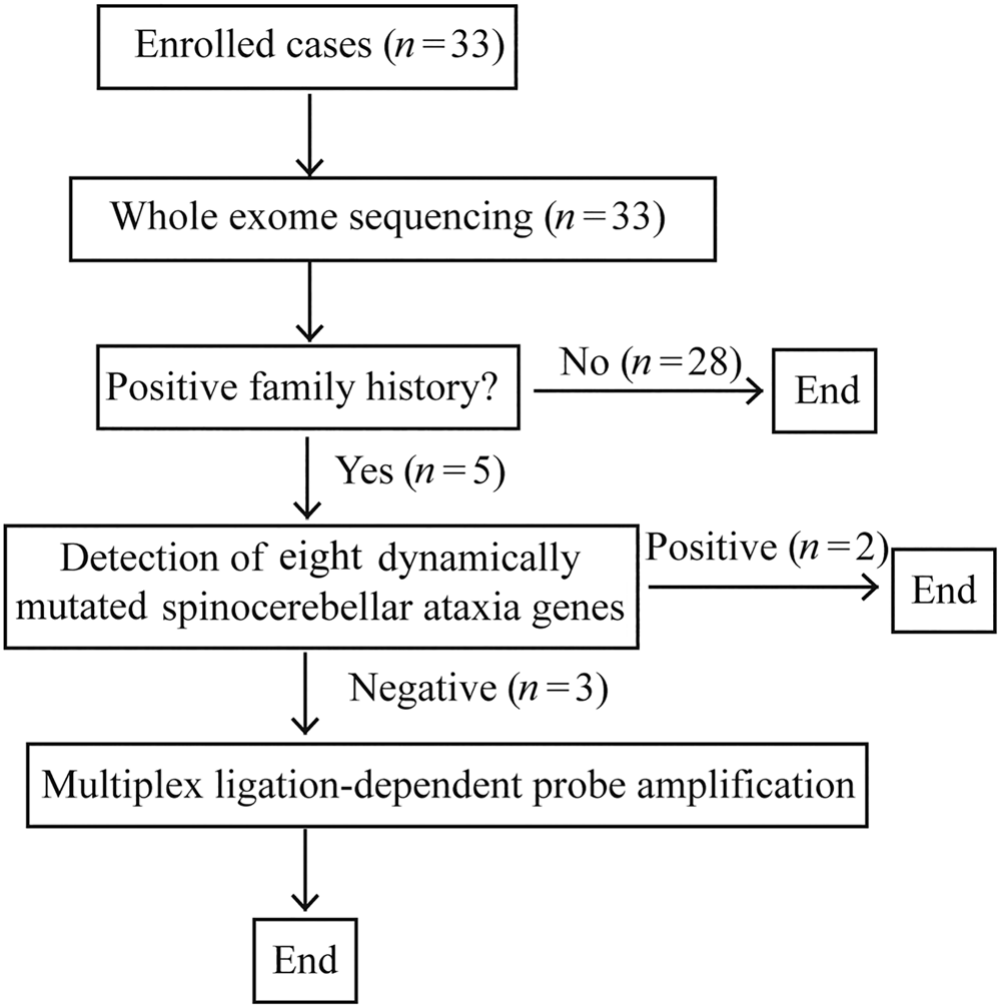
Illustration of the genetic testing process in this study.

Following informed consent, 5 mL of peripheral blood was collected from research subjects and normal controls to extract genomic DNA for genetic testing. The genetic testing process is illustrated in **Figure** 1.

### 2.3 WES Testing

Genomic DNA from all participants was extracted using the QIAamp DNA Blood Midi Kit (Qiagen, Hilden, Germany) following the manufacturer's standard procedures. The genomic DNA from the family was then fragmented using the Covaris LE220 (Massachusetts, USA) to generate a paired‐end library (200‐250 bp). The library was prepared using the SureSelectXT Human All Exon V8 kit (Agilent, USA) and enriched by array‐based hybridization according to the manufacturer's standard protocol, followed by elution and post‐capture amplification. The products were then assessed for enrichment on the Agilent 2100 Bioanalyzer and ABI StepOne. After quality control, the captured library was sequenced on an Illumina HiSeq X Ten analyzer (Illumina, San Diego, USA), following the manufacturer's standard sequencing protocols, with 150 cycles per read to generate paired‐end reads. Image analysis, error estimation, and base calling were performed to generate raw data using Illumina Pipeline software.

Bioinformatics processing and variant calling were performed on raw sequencing data. We used AfterQC to generate “clean reads” for further analysis. The “clean reads” (length of 150 bp) from the targeted sequence were subsequently aligned to the human genome reference (hg19) using Burrows Wheeler Aligner (BWA) software (Li and Durbin 2009). After alignment, the output files were used to perform sequencing coverage and depth analysis. Single‐nucleotide variants (SNVs) and indels were detected using the Genome Analysis Toolkit (GATK; https://software.broadinstitute.org/gatk).

Variant filtering and prioritization were performed as follows. Variants with a minor allele frequency (MAF) ≤ 0.005 in public databases, including the 1000 Genomes dataset, Genome Aggregation Database (gnomAD) v4, and the Exome Aggregation Consortium dataset (ExAC) (https://gnomad.broadinstitute.org/), were retained for further analysis. Functional effects of missense variants were predicted using the dbNSFP database. Variants were classified as damaging and potentially disease‐causing variants if they met any of the following criteria: (1) predicted loss‐of‐function variants, including stop‐gain, stop‐loss, frameshift indels, and canonical splice‐site variants; (2) deleterious missense variants with a REVEL score > 0.5; (3) splice‐disrupting variants with a SpliceAI delta score > 0.2; or (4) variants previously reported as pathogenic or likely pathogenic in the Human Gene Mutation Database (HGMD) (Stenson et al. 2020) or ClinVar database (Landrum et al. 2014). The pathogenicity of variants was assessed according to the protocols published by the American College of Medical Genetics and Genomics (ACMG) (Liu et al. 2016; Richards et al. 2015). All potential pathogenic variants were verified using traditional Sanger sequencing methods.

### 2.4 SCA Testing

Repeat mutations in SCA‐associated genes were detected using molecular genetic assays. Specifically, PCR was utilized to identify repeat expansions in *ATXN1, ATXN2, ATXN3, CACNA1A, ATXN7*, and *PPP2R2B*, while TP‐PCR was employed for the detection of *ATXN8* and *ATXN10*. The primer information used in this study is compiled in **Table** S1. After PCR amplification, capillary electrophoresis was carried out on an ABI3730 DNA Analyzer to analyze the length of the amplification products, and the number of tandem repeats was calculated using Gene Marker software.

### 2.5 MLPA Testing and Analysis

Copy number variations (CNVs) were detected in genes including *DJ‐1, ATP13A2, PINK1, UCHL1, SNCA, LRRK2, PRKN*, and *GCH1* using SALSA MLPA Probemixes (MRC Holland, Netherlands). The procedure and data analysis were performed according to the manufacturer's instructions. The main operational steps were as follows: (1) Denaturation: Based on the extracted sample genomic DNA concentration, 5 µL (total DNA amount of 100–200 ng) was diluted with the corresponding kit elution buffer, and the mixture was denatured at 95°C for 5 min. (2) Hybridization: The denatured genomic DNA was cooled to 25°C, and 1.5 µL of probes [P051/P052] were added, followed by 1.5 µL of MLPA buffer. The mixture was hybridized overnight at 60°C (approximately 16 h). (3) Ligation: The temperature was decreased to 54°C, 32 µL of ligation mixture (3 µL of Buffer A, 3 µL of Buffer B, 25 µL of H_2_O, and 1 µL of ligase‐65) was added, the mixture was incubated at 54°C for 15 min, and the ligation reaction was terminated at 98°C for 5 min. (4) PCR Amplification: The temperature was decreased to 25°C, and 10 µL of PCR mixture (7.5 µL of H_2_O, 2 µL of PCR primer mixture, and 0.5 µL of SALSA Polymerase) was added. The PCR amplification conditions were as follows: denaturation at 95°C for 30 s, annealing at 60°C for 30 s, and extension at 72°C for 1 min for a total of 35 amplification cycles, and extension at 72°C for an additional 20 min. (5) Capillary Electrophoresis Separation and Data Analysis: The PCR amplification products were separated by electrophoresis using an ABI 3500 capillary electrophoresis instrument. The peak patterns of the amplified products were observed, with the control group's detection results used as an internal control, and the detected raw results were analyzed using Coffalyser.Net software (MRC‐Holland, Netherlands).

### 2.6 Statistical Analysis

Data analysis was performed using SPSS 25.0 software. The measured data are expressed as the means ± standard deviations (mean ± SD) or medians (interquartile ranges), and the count data are expressed as counts (proportions).

## 3 Results

### 3.1 Demographic and Clinical Characteristics of the Patients

In this study, a total of 33 probands with YOPD were enrolled, including 15 females and 18 males. The mean age at assessment was 46.61 ± 6.26 years. The median age at disease onset was 44 years (IQR, 38.5–48), and the median disease duration was 3 years (IQR, 2–6). The median duration of education was 9 years (IQR, 6–12). Five patients (15.2%) had a positive family history of autosomal dominant inheritance (Patients 3, 8, 13, 28, and 29). Bradykinesia and/or rest tremor were the initial symptoms at diagnosis in 25 patients (75.8%). Rest tremor was observed in 18 patients (54.5%), and rigidity was present in 15 patients (45.5%). Additionally, six patients (18.2%) reported constipation, seven patients (21.2%) experienced hyposmia, and two patients (6.1%) presented with symptoms of rapid eye movement sleep behavior disorder (RBD). Twenty‐five patients completed the Unified Parkinson's Disease Rating Scale Part III (UPDRS‐III), with a mean score of 31.84 ± 17.40. The Mini‐Mental State Examination (MMSE) was administered to 23 patients (69.7%), yielding a median score of 27 (IQR, 22–29). In total, 30 patients (90.9%) showed a positive response to levodopa, whereas 3 patients (9.1%; Patients 3, 4, and 29) showed a poor response. Three patients underwent deep brain stimulation (DBS) during follow‐up (see **Table** 1 for details). **Patient 4** died in the seventh year of disease at age 48 years, and **Patient 3** died at age 34, 14 years after onset. **Patient 23** died at age of 47 years,5 years after disease onset, due to heart failure and multiple organ dysfunction.

**TABLE 1:** The clinical characteristics of the subjects in this study.

Pt No.	Gender	AAO (y)	DOI (y)	AAV (y)	Variant (Y/N)	FH	Tremor	Rigidity	Bradykinesia	Constipation	Hyposmia	RBD	UPDRS‐III	H‐Y	MMSE	Edu Yrs (y)	L‐Dopa resp.	MRI	SN‐STS
1	F	44	12	56	N	Neg	N	N	Y	N	N	N	65	3	27	4	Pos	NL	NA
2	F	49	1	50	Y	Neg	Y	Y	Y	N	N	N	NA	NA	NA	0	Pos	NL	L SN‐STS incomplete
3	F	20	8	28	Y	Pos	Y	N	N	N	N	N	49	3	NA	12	Neg	OPCA	NA
4	M	39	2	41	N	Neg	N	Y	Y	N	N	N	NA	NA	NA	12	Neg	CPA	NA
5	F	39	2	41	Y	Neg	N	Y	Y	N	N	N	NA	NA	NA	9	Pos	NL	B SN‐STS incomplete
6	M	45	6	51	Y	Neg	Y	Y	N	N	N	N	NA	NA	NA	12	Pos	NL	B SN‐STS incomplete
7	M	43	0.2	43	N	Neg	Y	N	N	N	N	N	NA	NA	NA	15	Pos	NL	L SN‐STS incomplete
8	M	36	4	40	N	Pos	Y	Y	N	N	N	N	40	2	20	15	Pos	NL	NA
9	M	35	5	40	Y	Neg	Y	Y	N	Y	N	N	34	2	29	16	Pos	NL	B SN‐STS incomplete
10	F	46	13	59	Y	Neg	Y	Y	Y	N	N	N	NA	NA	NA	0	Pos	NL	B SN‐STS incomplete
11	M	45	2	47	N	Neg	Y	N	Y	N	N	N	NA	NA	NA	9	Pos	NL	B SN‐STS incomplete
12	M	48	0.5	48	N	Neg	Y	N	Y	N	N	N	29	2	20	9	Pos	NL	NA
13	M	41	8	49	Y	Pos	Y	N	Y	N	Y	N	22	2	29	16	Pos	CPA	L SN‐STS incomplete
14	M	36	4	40	N	Neg	Y	N	N	N	N	N	35	2	27	9	Pos	NL	B SN‐STS incomplete
15	F	49	4	53	N	Neg	Y	N	Y	Y	Y	N	41	3	21	0	Pos	NL	B SN‐STS incomplete
16	F	33	16	49	N	Neg	Y	N	N	N	N	N	80	4	23	0	Pos	NL	NA
17	F	48	2	50	Y	Neg	Y	N	Y	N	N	N	16	1	29	12	Pos	NL	B SN‐STS incomplete
18	M	48	2	50	N	Neg	N	Y	Y	N	Y	N	21	2	28	9	Pos	NL	B SN‐STS incomplete
19	M	47	1	48	N	Neg	N	Y	Y	Y	Y	N	21	2	27	9	Pos	NL	B SN‐STS incomplete
20	M	49	3	52	N	Neg	Y	N	Y	N	Y	Y	6	1	26	9	Pos	NL	L SN‐STS incomplete
21	M	46	2	48	Y	Neg	N	Y	Y	N	N	N	23	1	29	9	Pos	NL	NA
22	M	49	6	55	N	Neg	Y	N	N	N	N	N	23	NA	21.5	6	Pos	NL	L SN‐STS incomplete
23	M	42	3	45	Y	Neg	Y	N	Y	Y	Y	Y	46	2.5	29	9	Pos	NL	B SN‐STS incomplete
24	M	44	3	47	Y	Neg	Y	N	Y	N	N	N	31	2	25.5	6	Pos	NL	L SN‐STS incomplete
25	M	48	0.5	48	N	Neg	N	N	Y	N	N	N	NA	NA	NA	12	Pos	NA	NA
26	M	38	2	40	N	Neg	N	N	Y	N	N	N	13	1	28	9	Pos	NL	B SN‐STS incomplete
27	F	44	5	49	N	Neg	N	Y	Y	Y	N	N	26	1	26	12	Pos	NL	B SN‐STS incomplete
28	F	43	6	49	Y	Pos	N	N	Y	N	N	N	37	2	18	0	Pos	NA	NA
29	F	25	10	35	Y	Pos	N	Y	Y	N	N	N	38	3	27	9	Neg	NL	B SN‐STS incomplete
30	F	48	3	51	Y	Neg	N	Y	Y	N	N	N	48	3	22	0	Pos	NL	B SN‐STS incomplete
31	F	45	3	48	Y	Neg	N	Y	Y	Y	N	N	15	1.5	NA	4	Pos	NL	B SN‐STS incomplete
32	F	32	9	41	N	Neg	N	Y	Y	N	Y	N	33	3	29	8	Pos	NA	NA
33	F	45	2	47	Y	Neg	N	N	Y	N	N	N	4	1	26	9	Pos	NL	NA

*Note*: Pt No.: patient number; AAO (y): age at onset (years); DOI (y): duration of illness (years); AAV (y): age at visit (years); Variant (y/n): genetic variants (yes/no); FH: family history; RBD: REM sleep behavior disorder; UPDRS‐III: Unified Parkinson's Disease Rating Scale Part III; H‐Y: Hoehn and Yahr stage; MMSE: Mini‐Mental State Examination; Edu, Yrs: years of education; L‐Dopa resp.: levodopa responsiveness, Pos: positive, Neg: negative; MRI: non‐contrast brain MRI findings; SN‐STS: substantia nigra swallow‐tail sign status; OPCA: olivopontocerebellar atrophy; CPA: cerebellar and pontine atrophy; M: male; F: female; N: no; Y: yes; NA: not available; NL: normal; L: left; R: right; B: bilateral.

### 3.2 Genetic Testing Results

#### 3.2.1 Pathogenic Variants and VUS in Known YOPD‐Associated Genes

According to the workflow shown in **Figure** 1, all 33 patients underwent WES, 5 cases underwent SCA testing, and 3 patients underwent MLPA testing and analysis. After variant filtering, a total of six variants in four known YOPD‐associated genes (*ATXN2, VPS13C, PRKN*, and *SNCA*) were identified in four patients. Among them, pathogenic *ATXN2* repeat expansions were detected in two patients (6.1%): **Patient 3** (CAG repeats 22/48) and **Patient 13** (CAG repeats 22/36). Four VUS (c.2111C>T and c.662C>T in *VPS13C*; c.1372A>C and c.1001G>A in *PRKN*) were previously documented in 1000 Genomes and gnomAD v4 databases. In addition, a heterozygous duplication involving exons 1–6 in *SNCA*, which was classified as VUS following assessment according to the ACMG CNV evaluation workflow, has also been reported in existing literature. All variants were validated by Sanger sequencing and subsequent family co‐segregation analysis (see **Table** **2** for details). Genetic findings in the mutation‐positive patients were individually summarized as follows: **Patient 3**, who carried the pathogenic *ATXN2* repeat expansion, was concomitantly found to have compound heterozygous variants (c.2111C>T and c.662C>T) in *VPS13C*; **Patient 13** was positive only for the pathogenic *ATXN2* repeat expansion; **Patient 22** harbored compound heterozygous variants (c.1372A>C and c.1001G>A) in *PRKN*, and **Patient 28** had a heterozygous duplication of exons 1–6 in *SNCA*.

**TABLE 2:** Genetic variants identified in known young‐onset Parkinson's disease genes.

Pt No.	Gender	AAO(y)	DOI (y)	AAV(y)	FH	Gene	MOI	Gene Transcript ID	Genomic Location (hg 19)	cDNA	AA	SNP No.	1000Genomes	gnomAD v4	SIFT	PolyP2	Func Pred	Path Class	ACMG	Func Valid
3	F	20	8	28	Pos	ATXN2(22,48)	AD	NM_001372574.1	chr12:111881289‐112037480	/	/	/	/	/	/	/	/	P	/	Y
						VPS13C	AR	NM_020821	chr15:62273596	c.2111C>T	p. T704M	rs114387705	0.00020	0.00003101	D	D	10.5/21	VUS	PM2	N
						VPS13C	AR	NM_020821	chr15: 62312700	c.662C>T	p. T221I	rs202137969	0.00020	0.000002482	D	D	11.5/21	VUS	PM2	N
13	M	41	8	49	Pos	ATXN2(22,36)	AD	NM_001372574.1	chr12:111881289‐112037480	/	/	/	/	/	/	/	/	P	/	Y
22	M	49	6	55	Neg	PRKN	AR	NM_004562.3	chr6:161771157	c.1372A>C	p. M458L	rs182893847	0.001	0.000176	T	B	13/21	VUS	PM2, PP3	N
						PRKN	AR	NM_004562.3	chr6:161969968	c.1001G>A	p. R334H	rs746215864	NR	0.00001549	T	B	5/21	VUS	PM2	N
28	F	43	6	49	Pos	SNCA dup(1‐6ex)	AD	NM_000345.4	chr4:90621496‐90759466	/	/	/	/	/	/	/	/	VUS	/	N

Pt No.: patient number; M: male; F: female; AAO: age at onset (y); DOI: duration of illness (years); AAV (y): age at visit (years); FH: family history (Pos = positive, Neg = negative); Gene: WES results (gene name); MOI: mode of inheritance; AD: autosomal dominant; AR: autosomal recessive; cDNA: nucleotide variation; AA: amino acid alteration; NR: not reported; SIFT/PolyP2: pathogenicity prediction software (B: benign; T: tolerated; A.P.F.: affect protein function; P.D.: possibly damaging); Func. Pred.: functional predictions (Deleterious/Total); Path Class: pathogenicity classification (VUS: variants of uncertain significance; P: pathogenic); ACMG: American College of Medical Genetics and Genomics classification; Func Valid: functional validation (N: no).

#### 3.2.2 Risk Variants in LRRK2

In contrast to the Mendelian pathogenic variants and VUS described in Section 3.2.1, we also assessed risk/susceptibility variants in *LRRK2*. Interestingly, in this study, we identified a considerable number of patients harboring risk variants in *LRRK2*, a gene associated with YOPD. The *LRRK2* c.7153G>A variant was identified in four patients (12.1%), and the c.4883G>C was identified in a separate set of four patients (12.1%), bringing the total number of patients carrying *LRRK2* risk variants to eight (24.2%). Among them, one patient also carried the c.5050C>T variant, as detailed in **Table** 3.

**TABLE 3:** Risk variants of *LRRK2* in patients with young‐onset Parkinson's disease.

Pt No.	Gender	AAO (y)	DOI (y)	AAV (y)	FH	Gene	Gene Transcript ID	Genomic Location (hg19)	cDNA	AA	SNP No.	1000Genomes	gnomADv4	SIFT	PolyP2	Func Pred	ACMG	Func Valid
5	F	39	2	41	Neg	LRRK2	NM_198578.4	chr12:40713845	c.4883G>C	p.R1628P	rs33949390	0.006	0.0009673	A.P.F.	P.D.	16.5/21	/	N
9	M	35	5	40	Neg	LRRK2	NM_198578.4	chr12:40713845	c.4883G>C	p.R1628P	rs33949390	0.006	0.0009673	A.P.F.	P.D.	16.5/21	/	N
						LRRK2	NM_198578.4	chr12:40714870	c.5050C>T	p.H1684Y	/	NR	NR	T	P.D.	10.5/23		N
14	M	36	4	40	Neg	LRRK2	NM_198578.4	chr12:40757328	c.7153G>A	p.G2385R	rs34778348	0.005	0.0007873	T	B	6/21	/	N
17	F	48	2	50	Neg	LRRK2	NM_198578.4	chr12:40757328	c.7153G>A	p.G2385R	rs33949390	0.005	0.0007873	T	B	6/21	/	N
18	M	48	2	50	Neg	LRRK2	NM_198578.4	chr12:40757328	c.7153G>A	p.G2385R	rs33949390	0.005	0.0007873	T	B	6/21	/	N
20	M	49	3	52	Neg	LRRK2	NM_198578.4	chr12:40713845	c.4883G>C	p.R1628P	rs33949390	0.006	0.0009673	A.P.F.	P.D.	16.5/21	/	N
29	F	25	10	35	Pos	LRRK2	NM_198578.4	chr12:40757328	c.7153G>A	p.G2385R	rs34778348	0.005	0.0007873	T	B	6/21	/	N
31	F	45	3	48	Neg	LRRK2	NM_198578.4	chr12:40713845	c.4883G>C	p.R1628P	rs33949390	0.006	0.0009673	A.P.F.	P.D.	16.5/21	/	N

*Note*: Pt No.: patient number; M: male; F: female; AAO (y): age at onset (y); DOI (y): duration of illness (years); AAV (y): age at visit (years); FH: family history (Pos: positive, Neg: negative); Gene: WES results (gene name); cDNA: nucleotide variation; AA: amino acid alteration; NR: not reported; SIFT/PolyP2: pathogenicity prediction software (B: benign; T: tolerated; A.P.F.: affect protein function; P.D.: possibly damaging); Func. Pred: functional predictions (Deleterious/Total); ACMG: American College of Medical Genetics and Genomics classification; Func Valid: functional validation (N: no).

#### 3.2.3 Variants in Susceptibility Genes Associated With YOPD

Among the 33 analyzed patients, nine unrelated patients carried nine variants in six susceptibility genes associated with YOPD. These nine variants — c.1054C>T in *EIF4G1*, c.823A>G in *COQ2*, four variants in *TENM4* (c.3053G>A, c.5012C>T, c.5279A>T, and c.7612C>T), c.1166C>T in *NR4A2*, c.1265G>A in *UQCRC1*, and c.1448T>C in *GBA1* were each identified in a single patient (see **Table** **4** for details).

**TABLE 4:** Variants in young‐onset Parkinson's disease‐associated susceptibility genes.

Pt No.	Gender	AAO (y)	DOI (y)	AAV (y)	FH	Gene	Gene Transcript ID	Genomic Location (hg19)	cDNA	AA	SNP No.	1000Genomes	gnomADv4	SIFT	PolyP2	Func Pred	ACMG	Func Valid
2	F	49	1	50	Neg	EIF4G1	NM_198242	chr3:184040176	c.1054C>T	p.R352W	rs765169450	NR	0.00003904	D	B	11.5/23	PM2	N
11	M	45	2	47	Neg	COQ2	NM_001358921.2	chr4:84188867	c.823A>G	p.T275A	rs769971059	NR	0.00001159	A.P.F.	B	18/22	/	N
12	M	48	0.5	48	Neg	TENM4	NM_001098816.3	chr11:78443446	c.3053G>A	p.R1018H	rs748691958	/	0.00006382	T	P.D.	16/22	PP3	N
14	M	36	4	40	Neg	NR4A2	NM_006186.3	chr2:157183425	c.1166C>T	p.A389V	/	0.0002	0.00004027	T	P.D.	12.5/23	/	N
15	F	49	4	53	Neg	TENM4	NM_001098816.3	chr11:78412646	c.5012C>T	p.A1671V	/	NR	0.0000006208	T	B	10/23	PM2	N
22	M	49	6	55	Neg	UQCRC1	NM_003365.3	chr3:48637533	c.1265G>A	p.R422H	rs754611181	NR	0.000009913	A.P.F.	P.D.	15/22	PM2	N
23	M	42	3	45	Neg	TENM4	NM_001098816.3	chr11:78387414	c.5279A>T	p.Y1760F	rs745395614	NR	0.00001498	A.P.F.	P.D.	19.5/23	PM2, PP3	N
29	F	25	10	35	Pos	TENM4	NM_001098816.3	chr15:62182359	c.7612C>T	p.R2538W	rs376296325	/	0.000127	A.P.F.	P.D.	18.5/22	PM2, PP3	N
30	F	48	3	51	Neg	GBA1	NM_000157.4	chr1:155205043	c.1448T>C	p.L483P	rs421016	0.003	0.001	A.P.F.	P.D.	18.5/21	LP (PS3, PM3, PP3)	N

*Note*: Pt No.: patient number; M: male; F: female; AAO (y): age at onset (y); DOI (y): duration of illness (years); AAV (y): age at visit (years); FH: family history (Pos = positive, Neg = negative); Gene: WES results (gene name); cDNA: nucleotide variation; AA: amino acid alteration; NR: not reported; SIFT/PolyP2: pathogenicity prediction software (B: benign; T: tolerated; A.P.F.: affect protein function; P.D.: possibly damaging); Func Pred: functional predictions (Deleterious/Total); ACMG: American College of Medical Genetics and Genomics classification; Func Valid: functional validation (N: no).

#### 3.2.4 Clinical Characteristics, Diagnostic Process, and Pedigree Features of Two Patients With Pathogenic ATXN2 Repeat Expansions

**FIGURE 2:**
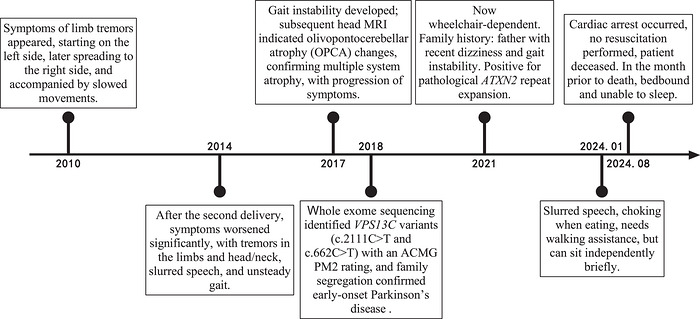
Timeline of medical history for Case 3.

**FIGURE 3:**
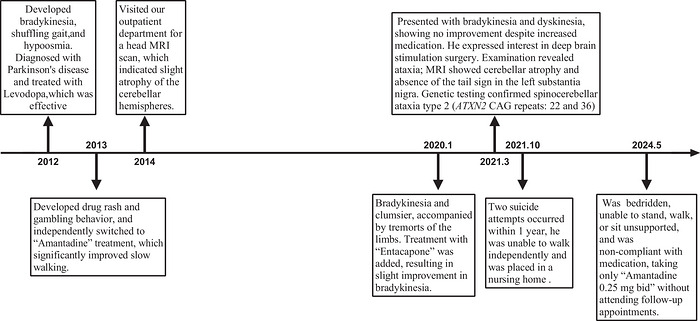
Timeline of medical history for Case 13.

Two patients with pathogenic *ATXN2* repeat expansion exhibited prominent positive family histories, with their detailed clinical courses summarized in **Figure** 2 and **Figure** 3, respectively.

**FIGURE 4:**
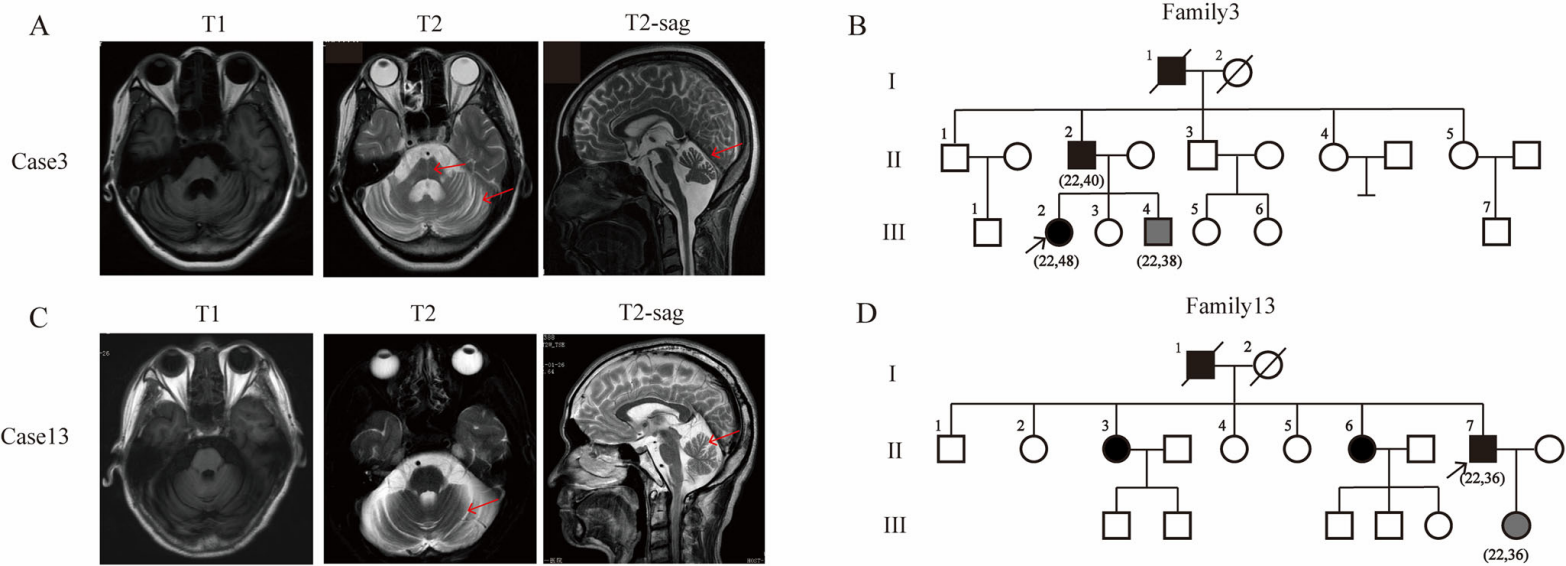
(A) Brain MRI of Case 3 showing brainstem and cerebellar atrophy (arrows). (B) Pedigree of Case 3. (C) Brain MRI of Case 13 showing brainstem and cerebellar atrophy (arrows). (D) Pedigree of Case 13. Pedigree legend—squares: males; circles: females; black symbols: affected subjects; white symbols: unaffected subjects; gray symbols: pre‐symptomatic individuals; arrows: proband.

**Patient 3** was a 28‐year‐old female who developed motor symptoms following pregnancy, with subsequent disease progression. She was initially misdiagnosed with multiple system atrophy (MSA) based on MRI findings suggestive of olivopontocerebellar atrophy (**Figure** 4A). *VPS13C* compound heterozygous variants were also identified as VUS, which initially confounded the diagnosis. Her UPDRS‐III score was 49 and Hoehn‐Yahr stage 3; however, treatment with levodopa, amantadine, trihexyphenidyl, buspirone, and clonazepam yielded no significant symptomatic improvement. The patient had multiple affected relatives, including her father, paternal grandmother, and great‐uncle (**Figure** 4B). Subsequently, a spinocerebellar ataxia (SCA) gene panel test was performed, which identified 48 CAG repeats in *ATXN2* (no CAA interruptions, see File S1), establishing a diagnosis of SCA type 2 (SCA2) after an 11‐year diagnostic delay. At the February 2024 follow‐up, she presented with severe ataxia and cognitive impairment, with the following scores: Scale for the Assessment and Rating of Ataxia (SARA) 34.5, International Cooperative Ataxia Rating Scale (ICARS) 93, Inventory of Non‐Ataxia Signs (INAS) 8, and MMSE 8. Her father (age at onset: 54 years old, 40 CAG repeats) exhibited gait instability, tremors, cognitive decline, and cerebellar ataxia with corresponding scale scores of SARA 10, ICARS 23, INAS 4, and MMSE 19. Her 30‐year‐old younger brother carried 38 CAG repeats and showed only mild nystagmus and subtle ataxia without overt symptoms with corresponding scale scores of SARA 0.5, ICARS 3, INAS 2, MMSE 27. **P** **atient** **3** died of sudden cardiac arrest in August 2024.

**Patient 13** was a 41‐year‐old male with an 8‐year history of progressive bradykinesia and tremors, initially diagnosed with PD at another hospital. Levodopa brought partial gait improvement, and entacapone was added 6 months pre‐admission for worsening motor symptoms. He was admitted to the hospital due to poor symptom control to assess his eligibility for DBS surgery. On physical examination, his vital signs were stable; he was alert and oriented with normal mental status. Neurological examination revealed bilateral finger‐nose ataxia, a positive Romberg sign, and impaired tandem gait. Cranial MRI showed cerebellar and pontine atrophy, while SWI demonstrated the disappearance of the swallow‐tail sign in the left substantia nigra (**Figure** 4C). No pathogenic variants were identified in common PD‐related genes by WES, but targeted testing detected 36 CAG repeats in *ATXN2* (no CAA interruptions, File S2), confirming SCA2 in the ninth year of his disease course. His family history was notable for PD in his father and two aunts (**Figure** 4D). His 20‐year‐old asymptomatic daughter was identified as a presymptomatic carrier of the identical pathogenic expansion (CAG 22/36), while other family members declined testing. Over time, the patient became bedridden with poor medication compliance, despite preserved cognition with corresponding scale scores of SARA 30, ICARS 61, INAS 10, and MMSE 25 at the 2024 follow‐up.

Collectively, these two patients with pathogenic *ATXN2* repeat expansions showed positive family histories and complex clinical courses, with long‐term misdiagnoses. Comprehensive clinical evaluation combined with targeted genetic testing for repeat expansions finally enabled an accurate diagnosis of SCA2. These cases illustrate that selecting the appropriate genetic testing approach is critical for achieving a precise diagnosis in clinically ambiguous patients.

## 4 Discussion

YOPD is a multifactorial disorder characterized by prominent genetic and clinical heterogeneity, and definitive genetic diagnosis for YOPD remains a major challenge to date. A considerable number of YOPD patients have not yet received an accurate clinical diagnosis. While genetic investigations into YOPD have been extensively carried out in Caucasian and Korean populations, such research remains limited in Chinese populations. In this study, WES and Sanger sequencing were performed on samples from all 33 YOPD patients of the Hakka population in western Fujian Province, with MLPA and SCA gene testing conducted in a subset of these patients. We detected *ATXN2* pathogenic repeat expansion in two patients and subsequently corrected their clinical diagnoses. Additionally, we identified four previously reported VUS including c.2111C>T and c.662C>T in *VPS13C*, c.1372A>C and c.1001G>A in *PRKN*, as well as a heterozygous duplication of exons 1–6 in *SNCA* (classified as a VUS).

In this study, we identified two SCA2‐positive patients (6.1%). International studies have shown a low frequency (1.7%–3.4%) of SCA2 positivity among patients with PD (Casse et al. 2023; Charles et al. 2007; Socal et al. 2009). Domestic studies on SCA2 prevalence in PD cohorts also reported low rates. One study reported two patients (1.5%) in a family with familial PD (FPD) and two individuals (0.5%) with *ATXN2* CAG repeats among 452 patients (386 with sporadic disease, 66 with familial disease) (Wang et al. 2009). Thus, the SCA2 detection rate in our study was notably higher than that previously reported in both domestic and international studies. SCA2 presents with diverse clinical features, such as ataxia, dysarthria, reduced tendon reflexes, and parkinsonism; this variability often leads to misdiagnosis. Both patients with SCA2 in our study experienced significant diagnostic delays (diagnosis at years 11 and 9 of the disease course in **Patient 3** and **Patient 13**, respectively). The proband from **Family 3** exhibited notable genetic anticipation, with her age at onset being 34 years earlier than that of her father; consequently, the father began to display symptoms in the 11th year of the proband's disease course, resulting in denial of family history at the proband's initial assessment and thus diagnostic difficulty. WES also revealed compound heterozygous *VPS13C* variants (c.2111C>T and c.662C>T) in **Patient 3**. Family segregation analysis revealed that the patient's father (II‐2) and sibling (III‐4) each carried only one *VPS13C* variant; consequently, the detection of *VPS13C* compound heterozygous variants (classified as VUS) created diagnostic confusion, contributing to the prolonged misdiagnosis. Early denial of family history, delayed accurate genetic testing, and marked anticipation all contributed to the late diagnosis for **Patient 3**. Notably, **Patient 3**’s disease was markedly more severe than that of her father and sibling. Although *VPS13C* VUS variants (c.2111C>T and c.662C>T) were identified in this patient, whether they contributed to the phenotypic severity remains unknown and requires further investigation.

Similarly, **Patient 13**’s diagnostic delay of 9 years (detailed in **Section** **3.2.4**) highlights the necessity for comprehensive SCA dynamic mutation testing in patients with familial PD. The high *ATXN2* positivity in this cohort is likely attributable to the small sample size; larger studies are warranted to determine whether unique mutational characteristics exist in this population. Together, the prolonged misdiagnoses in these two patients underscore the value of regular follow‐up and periodic updating of family histories, emphasizing the potential of comprehensive SCA testing to improve diagnostic accuracy.

In **Patient 28**, duplication of exons 1–6 in *SNCA* was detected, which was classified as a VUS per ACMG standards. Although *SNCA* duplications have been reported as pathogenic variants in the literature (Zhao et al. 2020; Nishioka et al. 2006), the VUS classification in the present case indicates that its pathogenicity has not been definitively established. **Patient 28**’s elder brother also carried the duplication but was asymptomatic. If the duplication is ultimately demonstrated to be pathogenic, this family pattern would be consistent with incomplete penetrance, observed to be approximately 50% in this family; comparable cases have previously been documented in the literature (Nishioka et al. 2006, 2009; Ahn et al. 2008). We identified a single patient with compound heterozygous *PRKN* variants (**Patient 22**), presenting with late onset at 49 years, no family history, a good response to levodopa, and slow disease progression. These compound heterozygous variants in *PRKN* were verified by family segregation analysis. Previous Chinese studies have revealed that *PRKN* variants are the most common pathogenic gene variants for YOPD (Lin et al. 2019; Zhao et al. 2020; Chen et al. 2022; Hua et al. 2022; Li et al. 2020).

Regarding *LRRK2*, no pathogenic (P) or likely pathogenic (LP) variants were found. However, *LRRK2* c.4883G>C and c.7153G>A variants were each detected in 12.1% of patients (four individuals each). It is important to clarify that these *LRRK2* variants are classified as risk/susceptibility factors rather than Mendelian pathogenic variants, and should be interpreted as such. *LRRK2* susceptibility variants should be interpreted with caution. Variants such as c.4883G>C and c.7153G>A are often classified as benign or have conflicting interpretations in ClinVar due to limited evidence of pathogenicity under ACMG criteria. Nevertheless, extensive population‐based studies support their role as genetic modifiers that increase disease susceptibility, particularly in Asian populations (Zheng et al. 2011; Tan et al. 2007; Xie et al. 2014; Zhang et al. 2009; Ross et al. 2008; Pan et al. 2023). For instance, G2385R, located in the WD40 domain, may affect *LRRK2* dimerization and promote apoptosis, while p.R1628P is part of an ancestral haplotype arising approximately 2500 years ago. Both variants have been consistently associated with increased PD risk in Chinese cohorts (Tan et al. 2007; Xie et al. 2014; Zhang et al. 2009; Ross et al. 2008; Pan et al. 2023). A study of *LRRK2* risk genes in 6995 white PD patients with 5595 controls, 1376 Asian patients with 962 controls, and 240 Arab‐Berber patients with 372 controls found that p.G2385R was associated with PD, while p.R1628P was not, likely driven by the predominance of white subjects in whom this variant is rare (Ross et al. 2011). Prior studies have shown that variants or their combinations in *LRRK2* affect kinase activity and risk variably (Tan et al. 2010). Another noteworthy finding is that the *LRRK2* c.4939T>A variant was detected in 51.5% of patients (17 patients) in our study cohort (**Table** S2). Previous studies have suggested that *LRRK2* c.4939T>A increases the risk of PD in southern China (Zheng et al. 2011). However, its high frequency in the present cohort (∼51%) raises the possibility that it may represent a common polymorphism in the Hakka population rather than a PD‐specific risk factor. Without a matched local control group, this finding cannot be interpreted as evidence of pathogenicity. Future studies incorporating ethnically matched controls from the same geographic region are needed to determine its relevance. Consistent with most studies in Chinese patients with PD, the LRRK2 p.G2019S variant, which is common in Europeans and Americans, was rarely detected (Tan et al. 2019). These findings confirm that the *LRRK2* variant spectrum differs significantly between ethnicities, emphasizing the importance of ethnicity‐specific genetic research.

*TENM4* variants were found in four patients, including one novel variant (p.A1671V) and one familial variant. The relationship between *TENM4* variants and YOPD remains debated. *TENM4* encodes a transmembrane protein implicated in neuropsychiatric disorders and was initially identified as a possible essential tremor (ET) gene in three families (Hor et al. 2015); subsequent studies from Canada and China did not support a direct link between *TENM4* and ET (Houle et al. 2017; Yan et al. 2020). However, a study of 1494 Chinese patients with YOPD and 1357 controls revealed that rare, loss‐of‐function or potentially damaging missense *TENM4* variants were associated with increased YOPD risk (Liang et al. 2021); another Chinese study reported that rare *TENM4* variants may increase PD risk and identified *TENM4* p.Y1760F as a potential pathogenic variant for YOPD (Pu et al. 2020). In our study, **Patient 23** (sporadic YOPD) tested positive for *TENM4* p.Y1760F, which is consistent with previous Chinese reports (Pu et al. 2020). Thus, our findings are consistent with the previously reported potential association between *TENM4* and YOPD (Liang et al. 2021; Pu et al. 2020), but larger samples and functional studies are needed to clarify the role of this gene.

In this study, **Patient 30** was identified as having the *GBA1* p.L483P variant, with an onset age of 48 years and rapid disease progression. By the third year of illness, the patient reached Hoehn and Yahr (H‐Y) stage 3 and currently exhibits significant motor fluctuations, with plans to undergo DBS surgery. Notably, the patient's MMSE score did not indicate any cognitive decline. This phenotypic presentation is consistent with the findings of previous studies examining YOPD within the Chinese population, indicating that the *GBA1* p.L483P variant is the most common variant, accounting for 40.51% of all variants (32 out of 79 alleles), with the vast majority of patients classified as having YOPD (Sun et al. 2023). Another study focusing on the Chinese population indicated that PD with *GBA1* variants (*GBA1*‐PD) is associated with accelerated motor and cognitive decline, particularly with more pronounced disabilities in bradykinesia, axial impairment, and visuospatial/executive function (Ren et al. 2023). Furthermore, large‐scale studies have shown that patients with YOPD carrying rare *GBA1* variants typically exhibit benign progression during the early stages of the disease, with no evidence of cognitive decline (Chen et al. 2020). The conclusions of the two studies differ for several reasons. The populations studied were distinct: the latter research was restricted to patients with YOPD, whereas the former included all patients with PD carrying *GBA1* variants. Additionally, there are variations in the clinical phenotypes associated with different *GBA1* variant sites. Of note, the single patient in our study (**Patient 30**) who carried the *GBA1* p.L483P variant presented with YOPD and rapid motor progression (reaching H‐Y stage 3 by year 3) but preserved cognition (MMSE within normal range). This pattern aligns with Ren et al.’s finding of rapid motor decline but is also consistent with Chen et al.’s observation of preserved cognition in early‐stage YOPD‐*GBA1*. Given that only a single case is available in our cohort, it is not possible to arbitrate between these two studies based on our data alone (Ren et al. 2023, 2024). However, given the limited sample size, this observation requires validation in larger cohorts.

Research on PD gene variants has deepened our understanding of disease mechanisms and has sparked considerable interest in gene therapy. Notable progress has been achieved; for example, an *LRRK2* inhibitor by Biogen/Denali (BIIB122) is in Phase III trials. Likewise, prasinezumab (an anti‐α‐synuclein antibody targeting *SNCA* aggregation—core pathology in PD, by Roche) is in Phase III, aiming to clear α‐synuclein aggregates. The NCT05287503 trial—a multicenter, randomized, double‐blind, placebo‐controlled study—is assessing whether high‐dose ambroxol can improve glucocerebrosidase activity and reduce central nervous system α‐synuclein, slowing cognitive and motor decline in 60 patients with PD who carry *GBA1* variants (Cavallieri et al. 2023). Thus, genetic testing for YOPD not only enhances clinical understanding and informs therapy selection but also provides essential references for future precision therapies and molecular interventions, with significant clinical and research implications.

## 5 Conclusion

This study elucidates the genetic mutation spectrum of YOPD among the Hakka population in western Fujian Province. A pathogenic ATXN2 repeat expansion was identified in two patients. Additionally, four VUS (in VPS13C and PRKN) and an SNCA exon 1–6 duplication (also classified as a VUS) were detected; the clinical significance of these VUS remains to be determined. We also detected three *LRRK2* risk loci associated with PD in the Chinese population and nine variants in six susceptibility genes related to YOPD. The relatively high prevalence of SCA2 in this cohort suggests that comprehensive clinical evaluation and genetic testing in early‐onset and familial PD patients may improve diagnostic accuracy and reduce misdiagnosis, thereby laying a foundation for more precise therapeutic strategies.

### 5.1 Limitations

Although we analyzed the genetic spectrum and clinical characteristics of patients with YOPD in the Hakka population of western Fujian Province, there are several limitations. First, while functional prediction software indicates the deleterious effects of rare variants and classifies them according to their potential pathogenicity per ACMG guidelines, additional experimental evidence is necessary to clarify their roles in PD. Second, although genotype‐phenotype analyses were performed in patients with and without pathogenic or likely pathogenic variants, further characterization is warranted due to the limited sample size.

## Author Contributions

**Li‐Ying Pan**: project administration, funding acquisition, writing – original draft, writing – review and editing, conceptualization. **Fang Guo**: investigation, formal analysis, data curation. Chong Zheng: investigation, data curation. **Xiao‐Hong Hu**: data curation. Yan‐Gui Chen: data curation, conceptualization. **Rong‐Rong Lin**: writing – review and editing, writing – original draft, formal analysis.

## Funding

This study was supported by a grant from the Fujian Province Natural Science Foundation to Li‐Ying Pan (2021J011434).

## Ethics Statement

The study was approved by the Ethics Committee of Longyan First Affiliated Hospital of Fujian Medical University (Approval No. [2021]014). Written informed consent for study participation and the publication of clinical details was obtained from all participants or their legally authorized caregivers.

## Conflicts of Interest

The authors declare no conflicts of interest.

## Supporting information




**Table S1**: Primer sequences for SCAs

**Table S2**: Risk Variants of *LRRK2* c.4939T>A in Patients with Young‐Onset Parkinson's Disease

**File S1**: Sanger sequencing chromatogram of the CAG repeat segment in *ATXN2* of Case 3

**File S2**: Sanger sequencing chromatogram of the CAG repeat segment in* ATXN2* of Case 13

## Data Availability

The original contributions presented in this study are included in the Supporting Information; further inquiries can be directed to the corresponding author.
