# Supplementary material for: Genetic Variants and Clinical Characteristics of Young‐Onset Parkinson's Disease in the Hakka Population of Western Fujian
Source: Brain Behav. 2026 May 27;16(6):e71504. doi: 10.1002/brb3.71504 (PMC13239422; doi:10.1002/brb3.71504)
Supplement: Supplementary file 1 — Table S1: Primer sequences for SCAs [file BRB3-16-e71504-s003.docx]

| **Table 1: Primer Sequences for SCAs** | |
| --- | --- |
| Primer Name | Primer Sequence |
| SCA1-F | FAM-GGGTGATGAGCCCCGGA |
| SCA1-R | GGAGGCCTATTCCACTCTGC |
| SCA2-F | FAM-TGCGAGCCGGTGTATGGG |
| SCA2-R | CGGGCGACGCTAGAAGGC |
| SCA3-F | FAM-CCAGTGACTACTTTGATTCG |
| SCA3-R | TGGCCTTTCACATGGATGTGAA |
| SCA6F | FAM-caaaagggtcagtCAGGTGTCCTATTCCCCTGTGATCCA |
| SCA6R | aaagggtcagtTGGGTACCTCCGAGGGCCGCTGGTG |
| SCA7F | FAM-gcggtcccaaaagggtcagtTGTTACATTGTAGGAGCGGAA |
| SCA7R | gtcccaaaagggtcagtCACGACTGTCCCAGCATCACTT |
| SCA8-F | FAM-CCCAATTCCTTGGCTAGACCC |
| SCA8-P3 | TACGCATCCCAGTTIGAGACG |
| SCA8-P4 | TACGCATCCCAGTTTGAGACGCAGCAGCAGCAGCAG |
| SCA10-F | FAM-CAGATGGCAGAATGATAAACTCAA |
| SCA10-P3 | TACGCATCCCAGTTTGAGACG |
| SCA10-P4 | TACGCATCCCAGTTTGAGACGAGAATAGAATAGAATAGAAT |
| SCA12-F | FAM-TGCTGGGAAAGAGTCGTG |
| SCA12-R | GCCAGCGCACTCACCCTC |
