# Supplementary material for: Genetic Variants and Clinical Characteristics of Young‐Onset Parkinson's Disease in the Hakka Population of Western Fujian
Source: Brain Behav. 2026 May 27;16(6):e71504. doi: 10.1002/brb3.71504 (PMC13239422; doi:10.1002/brb3.71504)
Supplement: Supplementary file 2 — Table S2: Risk Variants of LRRK2 c.4939T>A in Patients with Young‐Onset Parkinson's Disease [file BRB3-16-e71504-s004.docx]

**Supplemental Table 2:** **Risk Variants of *LRRK2* c.4939T>A in Patients with Young-Onset Parkinson's Disease**

| **Pt No.** | **Gender** | **AAO(y)** | **DOI (y)** | **AAV(y)** | **FH** | **Gene** | **Gene Transcript ID** | **Genomic Location (hg 19)** | **cDNA** | **AA** | **SNP No.** | **1000Genomes** | **gnomAD v4** | **SIFT** | **PolyP2** | **Func Pred** | **ACMG** | **Func Valid** |
| --- | --- | --- | --- | --- | --- | --- | --- | --- | --- | --- | --- | --- | --- | --- | --- | --- | --- | --- |
| 1 | F | 44 | 5 | 49 | N | LRRK2 | NM_198578.4 | chr12:40713901 | c.4939T>A | p. S1647T | rs11564148 | 0.3 | 0.2992 | T | B | 2/20 | / | N |
| 4 | M | 39 | 2 | 41 | N | LRRK2 | NM_198578.4 | chr12:40713901 | c.4939T>A | p. S1647T | rs11564148 | 0.3 | 0.2992 | T | B | 2/20 | / | N |
| 5 | F | 39 | 2 | 41 | N | LRRK2 | NM_198578.4 | chr12:40713901 | c.4939T>A | p. S1647T | rs11564148 | 0.3 | 0.2992 | T | B | 2/20 | / | N |
| 6 | M | 45 | 6 | 51 | N | LRRK2 | NM_198578.4 | chr12:40713901 | c.4939T>A | p. S1647T | rs11564148 | 0.3 | 0.2992 | T | B | 2/20 | / | N |
| 9 | M | 35 | 2 | 37 | N | LRRK2 | NM_198578.4 | chr12:40713901 | c.4939T>A | p. S1647T | rs11564148 | 0.3 | 0.2992 | T | B | 2/20 | / | N |
| 10 | F | 46 | 13 | 59 | N | LRRK2 | NM_198578.4 | chr12:40713901 | c.4939T>A | p. S1647T | rs11564148 | 0.3 | 0.2992 | T | B | 2/20 | / | N |
| 12 | M | 48 | 0.5 | 48 | N | LRRK2 | NM_198578.4 | chr12:40713901 | c.4939T>A | p. S1647T | rs11564148 | 0.3 | 0.2992 | T | B | 2/20 | / | N |
| 14 | M | 36 | 4 | 40 | N | LRRK2 | NM_198578.4 | chr12:40713901 | c.4939T>A | p. S1647T | rs11564148 | 0.3 | 0.2992 | T | B | 2/20 | / | N |
| 15 | F | 49 | 4 | 53 | N | LRRK2 | NM_198578.4 | chr12:40713901 | c.4939T>A | p. S1647T | rs11564148 | 0.3 | 0.2992 | T | B | 2/20 | / | N |
| 18 | M | 48 | 2 | 50 | N | LRRK2 | NM_198578.4 | chr12:40713901 | c.4939T>A | p. S1647T | rs11564148 | 0.3 | 0.2992 | T | B | 2/20 | / | N |
| 20 | M | 49 | 3 | 52 | N | LRRK2 | NM_198578.4 | chr12:40713901 | c.4939T>A | p. S1647T | rs11564148 | 0.3 | 0.2992 | T | B | 2/20 | / | N |
| 21 | M | 46 | 2 | 48 | N | LRRK2 | NM_198578.4 | chr12:40713901 | c.4939T>A | p. S1647T | rs11564148 | 0.3 | 0.2992 | T | B | 2/20 | / | N |
| 23 | M | 42 | 3 | 45 | N | LRRK2 | NM_198578.4 | chr12:40713901 | c.4939T>A | p. S1647T | rs11564148 | 0.3 | 0.2992 | T | B | 2/20 | / | N |
| 29 | F | 25 | 10 | 35 | Pos | LRRK2 | NM_198578.4 | chr12:40713901 | c.4939T>A | p. S1647T | rs11564148 | 0.3 | 0.2992 | T | B | 2/20 | / | N |
| 30 | F | 48 | 3 | 51 | N | LRRK2 | NM_198578.4 | chr12:40713901 | c.4939T>A | p. S1647T | rs11564148 | 0.3 | 0.2992 | T | B | 2/20 | / | N |
| 31 | F | 45 | 3 | 48 | N | LRRK2 | NM_198578.4 | chr12:40713901 | c.4939T>A | p. S1647T | rs11564148 | 0.3 | 0.2992 | T | B | 2/20 | / | N |
| 33 | F | 45 | 2 | 47 | Ng | LRRK2 | NM_198578.4 | chr12:40713901 | c.4939T>A | p. S1647T | rs11564148 | 0.3 | 0.2992 | T | B | 2/20 | / | N |
