# Supplementary figures and images for: Genetic Variants and Clinical Characteristics of Young‐Onset Parkinson's Disease in the Hakka Population of Western Fujian
Source: Brain Behav. 2026 May 27;16(6):e71504. doi: 10.1002/brb3.71504 (PMC13239422; doi:10.1002/brb3.71504)

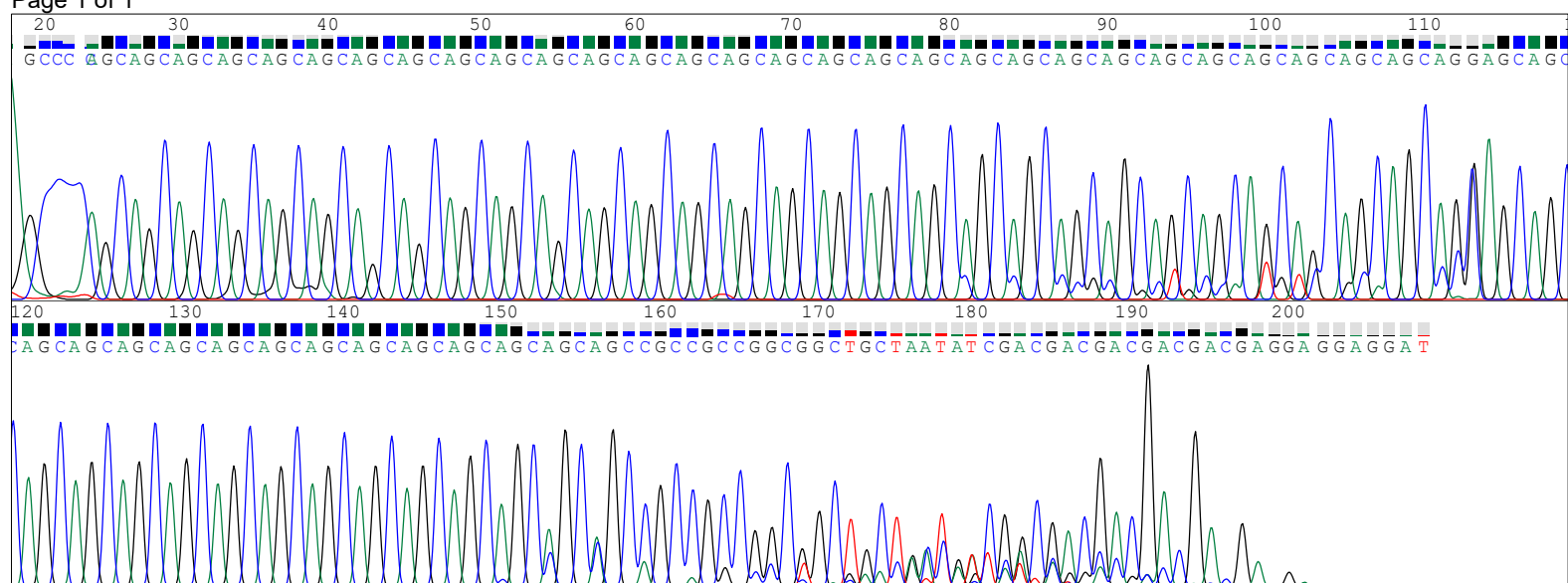

Supplement: Supplementary file 3 — File S1: Sanger sequencing chromatogram of the CAG repeat segment in ATXN2 of Case 3 [file BRB3-16-e71504-s002.pdf]

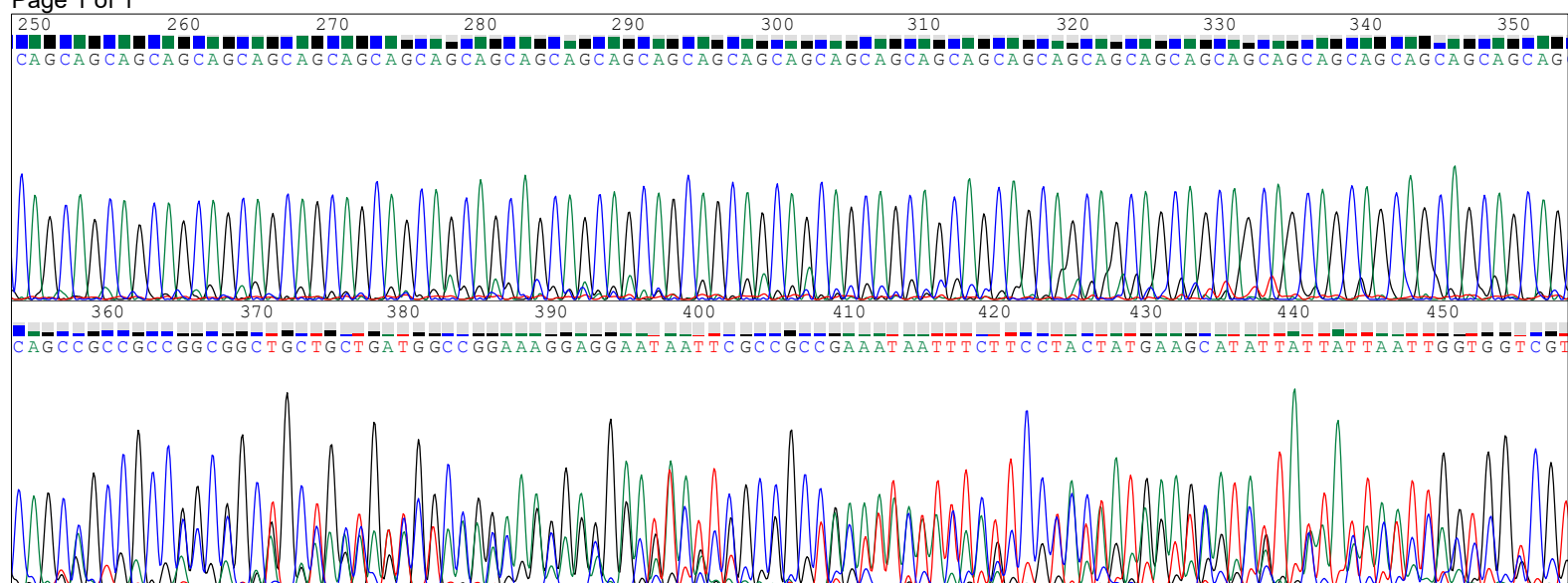

Supplement: Supplementary file 4 — File S2: Sanger sequencing chromatogram of the CAG repeat segment in ATXN2 of Case 13 [file BRB3-16-e71504-s001.pdf]
